# MagR Alone Is Insufficient to Confer Cellular Calcium Responses to Magnetic Stimulation

**DOI:** 10.3389/fncir.2017.00011

**Published:** 2017-03-16

**Authors:** Keliang Pang, He You, Yanbo Chen, Pengcheng Chu, Meiqin Hu, Jianying Shen, Wei Guo, Can Xie, Bai Lu

**Affiliations:** ^1^School of Pharmaceutical Sciences, Tsinghua UniversityBeijing, China; ^2^School of Life Sciences, Tsinghua UniversityBeijing, China; ^3^Tsinghua-Peking Center for Life Sciences, Tsinghua UniversityBeijing, China; ^4^Institute of Molecular Medicine, Peking UniversityBeijing, China; ^5^Artemisinin Research Center, Institute of Chinese Materia Medica, China Academy of Chinese Medical SciencesBeijing, China; ^6^School of Life Sciences, Peking UniversityBeijing, China

**Keywords:** magnetic field, calcium imaging, hippocampal neurons, neural modulation, cryptochrome, magnetogenetics

## Abstract

Magnetic manipulation of cell activity offers advantages over optical manipulation but an ideal tool remains elusive. The MagR protein was found through its interaction with cryptochrome (Cry) and the protein in solution appeared to respond to magnetic stimulation (MS). After we initiated an investigation on the specific role of MagR in cellular response to MS, a subsequent study claimed that MagR expression alone could achieve cellular activation by MS. Here we report that despite systematically testing different ways of measuring intracellular calcium and different MS protocols, it was not possible to detect any cellular or neuronal responses to MS in MagR-expressing HEK cells or primary neurons from the dorsal root ganglion and the hippocampus. By contrast, in neurons co-expressing MagR and channelrhodopin, optical but not MS increased calcium influx in hippocampal neurons. Our results indicate that MagR alone is not sufficient to confer cellular magnetic responses.

## Introduction

With the development and extensive use of optogenetics, neuroscience has made great strides, especially in behavioral and neural circuitry studies. The main advantage of light-gated ion channels, represented by the channelrhodopsin family (Boyden et al., 2005), is that they can be readily expressed in specific target brain regions or neuron types via a variety of genetics tools. Thus, the firing rate of channelrhodopsin-expressing neurons can be controlled by external light stimulation *in vivo* and *in vitro*. However, the drawbacks of optogenetics, such as the weak penetrating capability of light, the injury caused by optical fiber implantation, etc., are especially apparent when studying deep brain structures. More importantly, these drawbacks have made it difficult for human therapies. For instance, Parkinson's Disease is unlikely to be treated via channelrhodopsin expression coupled with optic fiber implantation for deep brain stimulation (Kringelbach et al., 2007).

Magnetic stimulation (MS) offers obvious advantages over light because of its deep penetration and non-invasiveness, if neurons could be made responsive to MS. One approach is the fusion of the ion-containing ferritin to mechano- or heat- sensing transient receptor potential cation channels, TRVP1 or TRVP4 (Stanley et al., 2016; Wheeler et al., 2016). However, the requirement of high magnetic field (50–500 mT) may limit its utility *in vivo*. Alternatively, a great deal of effort has been made to identify endogenous protein(s) that mediates magneto-reception in animals with geomagnetic sensitivity (e.g., pigeon and butterfly) capable of detecting the planet's weak magnetic field (around 50 micro-tesla). One such protein is MagR (also known as Iron-sulfur Cluster Assembly 1 or Isca1), which was identified as a putative magnetic receptor protein by Can Xie and colleagues (Qin et al., 2016). It was demonstrated that some 20 or so MagR molecules, when coupled with another protein chryptochrome (Cry), forms a multimeric rod-like protein complex capable of sensing and responding to magnetic fields *in vitro*. The possibility of the use of the MagR-containing protein complex in modulating neuronal activity—so called “magnetogenetics”—was raised (Qin et al., 2016). A theoretical physics calculation suggested that the number of iron atoms in the MagR/Cry complex may not be enough to sense magnetic fields (Meister, 2016). Thus, the physical principles and molecular mechanisms of MagR and MagR/Cry mediated magnetoreception remain unresolved.

We have been investigating whether expression of MagR could confer a neuronal response to MS. While our work was in progress, a report was published showing a robust increase in intracellular calcium in HEK 293 cells and hippocampal neurons transfected with MagR alone (in the absence of Cry), in response to weak MS (~1.0 mT) (Long et al., 2015). The audacious claim that this non-invasive approach may replace optogenetics for neural modulation has led to numerous attempts to replicate the work by researchers around the world, with no successful report thus far. In marked contrast to the published report, we could not detect any change in intracellular calcium induced by MS in cells expressing MagR alone. Thorough experiments were conducted using a number of different MagR constructs, two calcium image methods (GCaMP6 and Fura-2 AM) and three types of cells (HEK293, hippocampal neurons, and DRG neurons). Several different protocols of magnetic field stimulation (different directions, magnetic field power from 0.1 to 1.2 mT, and different time periods from a few seconds to 7 min) were used. All experiments included positive controls, and the experiments were repeated numerous times and in several different labs/rigs. All our attempts were to no avail. Our results demonstrate that MagR alone is insufficient to mediate cellular magnetic responses.

## Materials and methods

### Plasmid construction

Pigeon MagR cDNA was provided by C. Xie (Peking University). GCaMP6s was obtained from Addgene. RCaMP was obtained from Dr. Zhuan Zhou of Peking Univ. MagR was cloned into either AAV vector by PCR. In some experiments, MagR was linked to GCaMP6s by P2A nucleotide sequence through its N-terminus (pAAV-EF1α- GCaMP6s-P2A-MagR). In others, MagR was linked to mCherry by IRES sequence plenti-EF1α-MagR-IRES-mCherry-3flag). These constructs were created in several steps using PCR methods. Plasmids were confirmed sequencing of all cloned fragments in each step.

### Cell culture and transfection

HEK293A and HEK293T cells were maintained in high-glucose DMEM (Dulbecco's Modified Eagle Medium, Life Tech) with 10% fetal bovine serum (FBS, Life Tech) and 1% GlutaMAX-I (Invitrogen). Cells were plated on 18-mm poly-D-lysine–coated coverslip at 80,000 cells per well in a 12-well plate for calcium imaging. After overnight incubation, cells were transfected with various constructs using Lipofectiom-2000 (Life Tech).

### Western blotting

Western blot analysis was used to determine the level and the intactness of the MagR protein in cells transfected with MagR constructs. HEK293 cells were transfected with MagR-mCherry, and 24 h after transfection, the cells were lysed in buffer containing 50 mM Tris-HCl (pH 8.0), 250 mM NaCl, 1% NP-40, 0.5% deoxycholate, 0.1% SDS, and protease inhibitors (Roche Diagnostics). After centrifugation to remove insoluble material, the proteins in in lysate were separated using10% SDS–PAGE, and transferred to a PVDF membrane (Immobilon-P, Millipore). Membrane was blocked with 5% BSA in Tris buffered saline with 0,1% Tween (TBST) and incubated overnight at 4°C with Anti-MagR monoclonal antibody(1:500) diluted in 5% BSA in TBST, with gentle shaking. Membranes were washed with TBST, incubated with secondary antibodies (Goat Anti-Mouse, Thermo, 1:5,000), washed first with TBST and then with TBS, and developed with SuperSignal West Pico Chemiluminescent substrate (Pierce).

### Immunostaining

HEK293 cells were fixed for 30 min in pre-warmed phosphate buffer saline (PBS) with 4% paraformaldehyde at 37°C, permeabilized with PBS containing 0.1% Triton X-100 (30 min, 37°C), and then treated with blocking buffer (5% goat serum, 0.05% Tween20, PBS) for 2 h at room temperature. The cells were incubated with anti-MagR monoclonal antibody (#44–144, 1:500, diluted in blocking buffer) overnight at 4°C. Next day, the cells were rinsed 3 times in PBS, and exposed to Alexa Fluor®647 donkey anti-mouse IgG (1:500, Invitrogen, Carlsbad, CA) or Alexa Fluor®594 goat anti-mouse IgG (1:500, Invitrogen, Carlsbad, CA) secondary antibodies for 1 h in a dark chamber followed by counterstaining with 10 μg/ml DAPI for 10 min at room temperature. Finally, the cells were mounted using Vectorshield mounting media (Vector, Burlingame, USA) and viewed using Nikon laser scanning confocal microscopy. Imaging in sequential scan mode with 405, 488, 594, and 640 nm laser lines and customized filters were used for detection of different fluorophores. Images were prepared using Imaris software.

### Primary neuron culture and transfection

All animal experiments were carried out in accordance with the recommendations of AAALAC (Association for Assessment and Accreditation of Laboratory Animal Care International). The IACUC (Institutional Animal Care and Use Committee) of Tsinghua University approved all animal protocols (16-LB3) used in this study. The pregnant rats were euthanized following IACUC protocol. Rat hippocampal neurons (embryonic day 18) were dissociated with 1 ml 0.25% trypsin (1:1, Life Tech) in Hank's Balanced Salt Solution (HBSS, Life Tech) at 37°C. After 30 min incubation, the enzyme solution was removed and washed in warmed DMEM, with 10% FBS added to stop the enzymatic digestion. Cells were then plated on 18-mm poly-D-lysine–coated coverslip at 250,000 cells per well in 24-well plates. After overnight incubation, the culture medium was replaced with NeuroBasal medium (Invitrogen) with 2% B-27 (Invitrogen) and 1% GlutaMAX-I (Invitrogen). Neurons were transfected with various constructs at 6–10 DIV using calcium phosphate (Jiang and Chen, 2006).

### DRG neuron preparation

We prepared freshly isolated DRG neurons following the method described in Huang and Neher (1996) with slight modifications. The use and care of animals in this study followed the guidelines of the Peking University. Briefly, DRG of both cervical and lumbar spinal cord were taken from 14 to 18 day-old Sprague-Dawley rats. The surrounding connective tissue sheath was removed and the remaining tissues were digested with 1.5 mg/ml collagenase D (Boehringer Mannheim) and 0.2 mg/ml trypsin (GIBCO) at 35°C. Neurons were dissociated by trituration in culture medium (50% DMEM 10% F12) containing 50 mg/ml DNase. Collected dissociated cells were transfected with GCaMP6s and MagR-mCherry by electroporation, then plated on coverslips. Plating medium was replaced by fresh culture medium 15-20 min later. The experiments were carried out within 24 h after plating.

### Magnetic stimulation

A homemade device created by the Xie lab was used to deliver MS. The device consists of two pairs of coils arranged perpendicularly, with each coil pair aligned to generate magnetic fields with same polarity. The coils are connected to a controller, which allows direct current to pass through either pair of coils, and the amplitude could be adjusted. We placed 3.5 mm petri dishes in the center of the device, surrounded by the coils. A probe of a gaussmeter (WT10A, teslameter, WEITE MAGNETIC TECHNOLOGY CO., LTD) was placed to the center of culture dish very close to the imaged cells on the microscopic stage to measure the strength of MS applied to the cells. When turned on, the field strength at the center of the dish reached 1.2 mT. To generate a much stronger static magnetic field, we used a neodymium-iron-boron permanent magnet (D 40 mm × 20 mm each, axially magnetized, Hongfeng Magnets, Shanghai, China). This magnet could produce a magnetic flux density over 400 mT at the magnet surface. Field strengths of 150 mT at the center of the culture dish were generated by moving the magnet close to the culture dish from above by an electrically controlled retractable manipulator (Max distance is 100 mm, and the speed is 90 mm/s).

### Optic stimulation

Cultured hippocampal neurons were co-transfected with ChR2-YFP-P2A-MagR and RCaMP (Akerboom et al., 2013). The presence of YFP indicates the expression of ChR2 and MagR. Co-transfected cells were then subjected to MS using homemade coils as described above. After the magnetic field stimulation was turned off, 473 nm laser was delivered by optical fiber connected to a laser generator, which was controlled by Master-8 pulse stimulator. RCaMP (excitation wavelength 510 nm) was used instead of GCaMP6 (excitation wavelength 488) as a calcium indicator because optic stimulation laser (473 nm) may interfere with GCaMP6 imaging. RCaMP fluorescence was monitored during the whole experiment.

### Calcium imaging

Hippocampal neurons and HEK293A cells expressing GCaMP6 or RCaMP were subjected to live cell imaging following conventional procedures (Akerboom et al., 2013). Briefly, the culture medium was replaced by HEPES buffer (HEPES in HBSS, pH7.2) 30 min before imaging. Image series were acquired using an inverted microscope (Zeiss) or a 2-photon microscope (Olympus) at 2 Hz, and were processed with ImageJ afterwards. For HEK293A images, ROIs were defined by manually drawing ellipsoid areas that enclosing individual cells. For hippocampal neuron images, the frame with highest intensity of each series (3–5 frames after adding potassium chloride) was selected so that the whole cell area was clearly visible. A threshold was set and then the frame was converted to a binary mask where the cell areas were foreground objects. ROIs were defined by the outline of the foreground objects in each mask (Burger and Burge, 2008). The integral fluorescent intensity within each ROI was measured in all frames, and the raw intensity of the frames before stimulation onset was averaged and set as F_0_. The relative change in fluorescent intensity (ΔF/F_0_) of the ith frame could be calculated as (F_*i*_ – F_0_)/F_0_. We then plotted the ΔF/F_0_ against the elapsed time.

### Fura-2 single-cell Ca^2+^ imaging

Transfected HEK-293A cells and postnatal day 7 (DIV7) rat hippocampal neurons grown on coverslips were loaded with ratiometric Ca^2+^ indicator dye Fura-2 (Molecular Probes) (Final concentration 2.5 μg/mL) in the Ca^2+^ imaging buffer (1× Hanks Balanced Salt Solution (HBSS, 1.3 mM Ca^2+^) supplemented with 10 mM HEPES) for 30 min at 25°C and then subject to imaging on a Nikon ECLIPSE Ti-E microscope (×20 objective). The intracellular Ca^2+^ concentration was expressed as the 340/380 ratio and recorded as the ratio at each time point. Data are collected by MetaFluor (Molecular Devices, LLC), and processed with GraphPad Prism 6.0.

## Results

### Lack of Ca^2+^ responsiveness to magnetic stimulation in cell lines expressing MagR alone

Even before the publication of the paper describing the sequence and physicochemical properties of MagR (Qin et al., 2016), Long et al. published a paper reporting that magnetic field stimulation (MS) could induce a robust calcium influx in mammalian cells expressing the pigeon MagR (Long et al., 2015). Given its potential significance, it is important that the findings be replicated and validated by other laboratories. We had initiated this line of research much earlier, in January 2015, and addressed this issue in a systematic way using multiple approaches, constructs, cell types, techniques and methods.

In the first series of experiments, we transfected a human embryonic kidney (HEK)-derived cell line (293A) with GCaMP6-P2A-MagR, a plasmid expressing GCaMP6 and MagR linked by a self-cleaving peptide (P2A) in order to ensure co-expression of MagR and GCaMP6 in the same cells. GCaMP6 was used to monitor changes in intracellular calcium levels. The cells and the construct were similar to those used in the previous report (Long et al., 2015). MS was applied to the cells through a homemade device designed and fabricated by Dr. Can Xie containing two pairs of perpendicularly arranged coils (the same as the one used by Long et al., 2015). Each was powered by direct, adjustable currents, generating a static magnetic field of 0.1–1.2 mT on the cells recorded. The strengths of the magnetic fields at the center of the culture dish were monitored using a gaussmeter.

**Figure 1:**
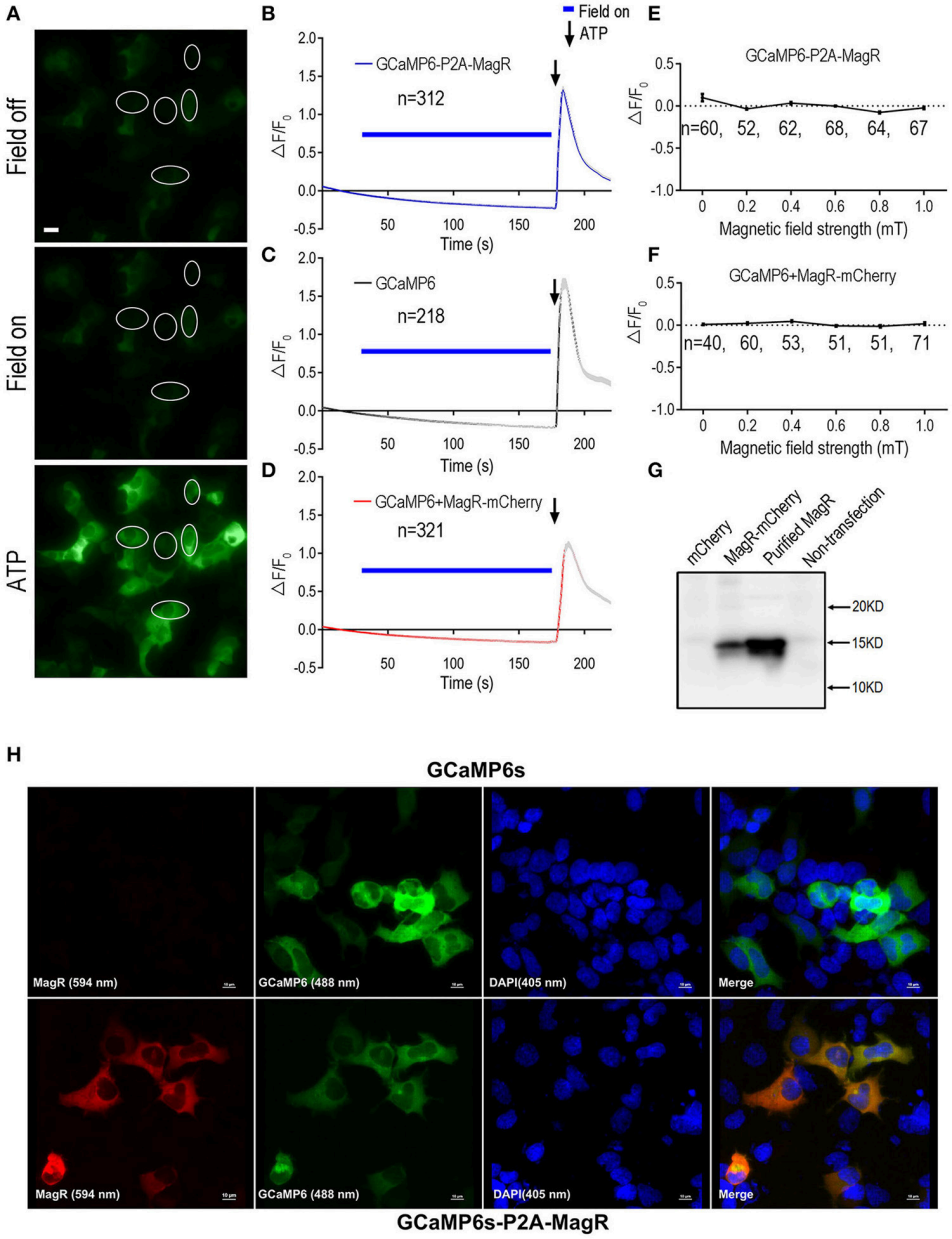
**GCaMP6-based Ca^**2+**^ imaging in MagR-expressing 293 cells**. Fibroblast HEK293A cells were transfected with either a plasmid in which MagR and GCaMP6 were linked by P2A sequence (GCaMP6-P2A-MagR), or co-transfected with MagR-mCherry and GCaMP6. On the next day, cells were subjected to magnetic field stimulation while Ca^2+^ changes were monitored by epifluorescence imaging. ATP was applied at the end of experiments to verify the viability of transfected cells and the ability of GCaMP6 to monitor changes in intracellular Ca^2+^. **(A)** Representative images showing the fluorescent intensity of HEK293 cells before (Field off) and during (Field on) magnetic stimulation, and a few seconds after applying ATP (ATP). Scale bar: 20 μm. **(B–D)** Quantification of the fluorescent intensity changes (ΔF/F_0_) in 293 cells transfected with the indicated constructs over time upon 1.0 mT magnetic field stimulation. Magnetic stimulation was indicated by blue bars above the curve, ATP was applied after switching off the magnetic field (arrows). The gradual decline of ΔF/F_0_ over time was due to photo bleaching. “n” indicates the number of cells recorded. **(E,F)** Stimulation-response curves. Cell responses were determined under different magnetic field strengths. ΔF/F_0_ at 27 s. after switching on of magnetic field was plotted against field strengths. Linear regression models show no correlation between MS field strengths and ΔF/F_0_ (For 1E, y = −0.11x (*r*2 = 0.46; for 1F, y = −0.09x (*r*2 = 0.089). The slopes of both curves were not significantly different from zero [*F*_(1, 4)_ = 3.36, *p* = 0.14, and *F*_(1, 4)_ = 0.39, *p* = 0.57, respectively]. No changes were observed for magnetic field stimulation up to 1.0 mT. In this and all other figures, data are presented in Mean ± SEM. **(G)** Western blot showing the expression of MagR protein in transfected cells. HEK293 cells were transfected with MagR-mCherry. A monoclonal antibody specific for MagR was used to detect MagR expression. Purified recombinant MagR is used as a positive control and lysates from cells not transfected or transfected with mCherry alone were used as negative controls. **(H)** Immunostaining showing co-expression of MagR and GCaMP6s proteins in transfected cells. HEK293 cells transfected with GCaMP6s (upper panels) or GCaMP6-P2A-MagR (lower panels), and immunostained with a mouse monoclonal anti-MagR antibody, followed by Alexa Fluor®594 goat anti-mouse IgG secondary antibody (excitation wavelength 594 nm). GCaMP6 was excited at 488 nm. The cells were also nuclear stained with DAPI (405 nm). Merge views show co-localization of MagR and GCaMP6s. Scale bar = 10 μm.

In contrast to the report by Long et al. who observed a 350% increase in the GCaMP6 florescence corresponding to a rise in intracellular calcium upon a brief magnetic field stimulation (Long et al., 2015), we observed no change in GCaMP6 fluorescence signal after the application of the magnetic field (Figures 1A,B). The weak fluorescence of GCaMP6 in cells before MS suggest that GCaMP6-P2A-MagR was expressed and cleaved successfully (Figure 1A). The magnetic field was applied in various lengths, up to 150 s. Instead of an increase in intracellular calcium, we observed a trend of decrease in calcium signals due possibly to photo-bleaching (Figure 1B). Extracellular ATP is known to induce calcium influx in these cells through adenosine P2X receptor, or trigger calcium release from endoplasmic reticulum through P2Y receptor (Glaser et al., 2013). We therefore applied ATP (500 μM) a few seconds after the MS was turned off. A dramatic increase in calcium signal was observed, indicating that these cells were healthy and can exhibit changes in intracellular calcium (Figures 1A,B). As a negative control, cells transfected with GCaMP6 alone also exhibited no response to MS, and application of ATP also induced a robust calcium response (Figure 1C).

We next examined whether magnetic field of different strengths could alter intracellular calcium concentration ([Ca^2+^]i). The change of GCaMP6 fluorescence intensity (ΔF/F_0_) at 27 s after turning on of magnetic field was plotted against the strengths of MS at the center of the culture dish. No change in [Ca^2+^]i was observed in response to magnetic field stimulation from 0 to 1.0 mT (Figure 1E).

The N-terminus of MagR is required for its interaction with the Cry protein (Qin et al., 2016) and perturbation at MagR N-terminus attenuated its ability to respond to the magnetic field (Xie, unpublished observations). We therefore transfected two separate plasmids, GCaMP6, and MagR-IRES-mCherry, instead of GCaMP6-P2A-MagR, into the 293A cells. An IRES was inserted in between MagR and mCherry. This allows the translation of mCherry from the initiation site IRES on the MagR-IRES-mCherry mRNA, independent of MagR translation, and therefore generating MagR and mCherry (as a marker of transfected cells) proteins separately in the same cells. Approximately 93% co-localization GCaMP6 (emission fluorescent at 510 nm) and MagR-mCherry (emission fluorescence at 610 nm) was achieved (data not shown).

Again, application of magnetic field up to 120 s induced no change in ΔF/F_0_ in the co-transfected cells (Figure 1D). We also applied magnetic fields of different strengths. Essentially same results were obtained: the GCaMP6 and MagR co-transfected cells showed no increase in intracellular calcium level from 0 to 1.0 mT (Figure 1F). We further increased the strength of MS up to 10.0 mT or used handheld magnetic bar, and still no response was observed (data not shown). Further, we performed the same experiments using 293T instead of 293A cells, which has a better attachment to the culture dishes. Again, we observed no change in intracellular calcium when MS was applied to the cells (data not shown). Taken together, these results indicate that application of magnetic field to cells expressing MagR does not induce intracellular calcium changes, regardless of the strengths or duration of the MS, or the cell line used.

To determine whether the MagR and GCaMP6 pair, or the GCaMP6 and MagR-mCherry pair, were expressed in the same cells, we performed confocal imaging experiments using cells immunostained by a newly generated monoclonal antibody against MagR. In cells transfected with GCaMP6-P2A-MagR, the MagR immunofluoscence (red) and GCaMP6 fluorescence (green) were completely overlapping, suggesting that the two proteins are co-expressed (Figure 1H, lower raw). As a control, cells transfected with GCaMP6 only exhibited GCaMP6 fluorescence but no MagR immunostaining (Figure 1H, upper raw). Similarly, in MagR-IRES-mCherry and GCaMP6 co-transfected cells, the MagR (purple), mCherry (red) and GCaMP6 (green) were co-localized in the same cells, whereas no MagR signal was detected in cells transfected with GCaMP6 only (Supplemental Figure 1). We next performed Western blots to determine whether intact MagR was expressed in these cells. As shown in Figure 1G, a MagR-specific monoclonal antibody detected a single band of 15 KD, exactly the same as the purified recombinant MagR, in cells co-transfected with GCaMP6 + MagR-IRES-mCherry. No signal was detected in non-transfected cells or cells transfected with mCherry alone (Figure 1G). These results together suggest that MagR and GCaMP6 co-exist in the same cells, and MagR and mCherry were translated independently and both were expressed well.

Finally, as a negative control, we used the HEK293T cells without exogenous MagR. In a few out of hundreds of 293T cells transfected with only GCaMP6 but no MagR, we saw some sporadic increases in Ca^2+^ fluorescence (Supplemental Figure 2, 4 examples). There was no obvious correlation between the Ca^2+^ signals and “on” or “off” of the MS, or the direction of the MS (X- axis or Y-axis). Occasionally, an increase in ΔF/F_0_ was observed in these cells expressing no MagR (e.g., green line). Since the Ca^2+^ signal did not correlate with MS, we next removed the magnetic field altogether. The sporadic Ca^2+^ responsiveness was still observed occasionally (Supplemental Figure 3). These results demonstrated that the sporadic fluctuation of intracellular Ca^2+^ could be observed even without MagR, and caution must be exercised not to take the spontaneous changes in Ca^2+^ concentrations as a magnetic response mediated by MagR.

### Lack of Ca^2+^ responsiveness to magnetic stimulation in neurons expressing MagR alone

**Figure 2:**
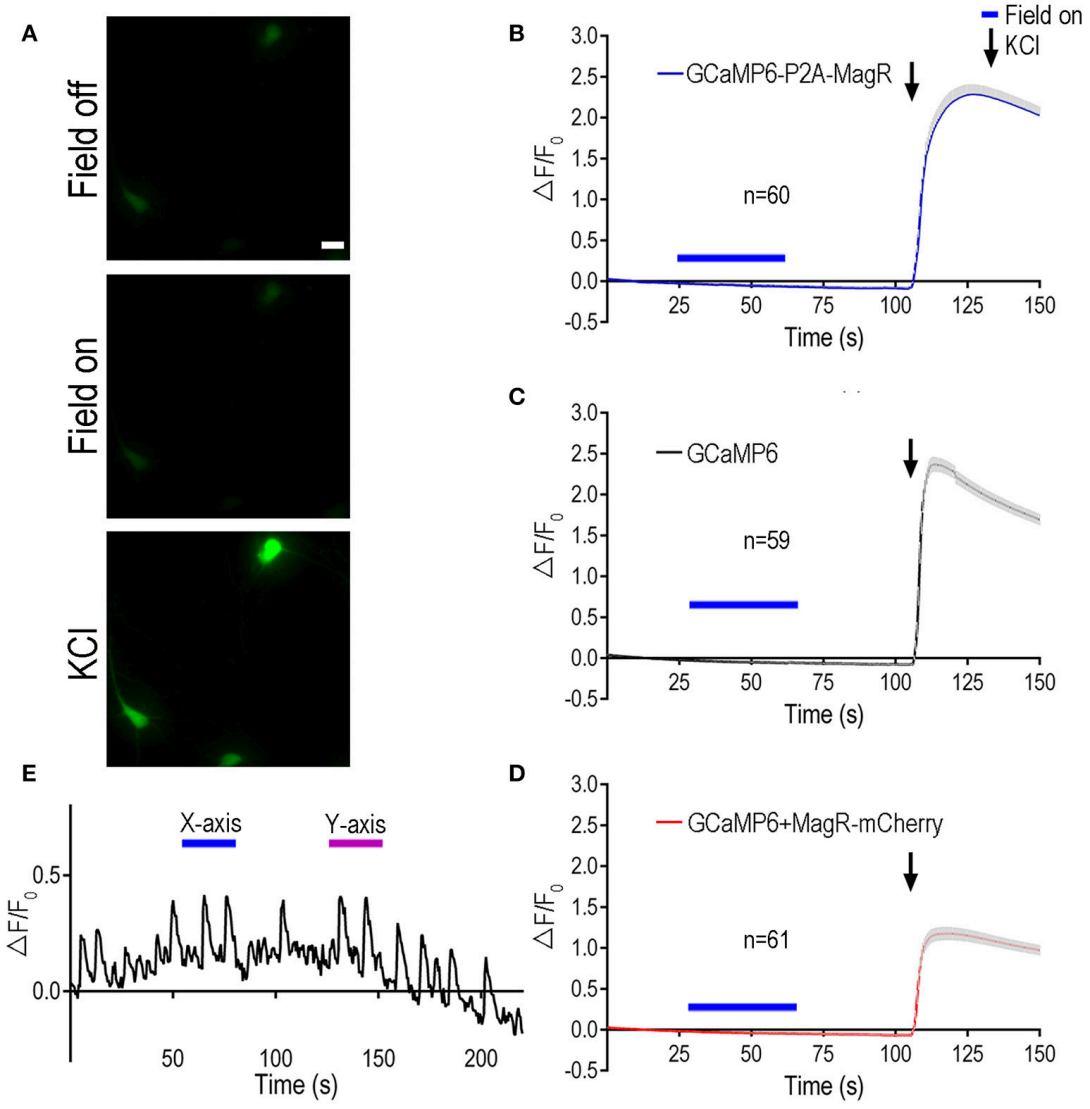
**Magnetic stimulation unable to induce Ca^**2+**^ change in hippocampal neurons expressing MagR**. All procedures were the same as Figure 1 except hippocampal neurons cultured for 6–10 days before DNA transfection, and high KCl (50 mM) was applied at the end of experiments. **(A)** Representative images showing the fluorescent intensity before (upper) and during (middle) magnetic stimulation, and a few seconds after applying high KCl (lower). Scale bar: 20 μm. **(B–D)** Quantification of the fluorescent intensity (ΔF/F_0_) changes over time upon 1.0 mT magnetic field stimulation. While magnetic stimulation failed to induce any changes in intracellular calcium, KCl elicited dramatic increase in ΔF/F_0_, indicating normal neuronal Ca^2+^ influx. Magnetic stimulation was indicated by blue bars above the curve, KCl application was indicated by arrows. **(E)** Examples of intensity plots from neurons which underwent spontaneous oscillations over time. Blue bars indicate application of magnetic field in one direction (termed X-axis), while magenta bars refer to magnetic field in another, perpendicular direction (termed Y-axis).

HEK 293 cells lack the cellular components required for excitability. To examine the role of MagR in excitable cells such as neurons, we transfected primary rat hippocampal neurons with MagR and GCaMP6, and applied magnetic field stimulation following the procedure described above (Figure 2). The previous report had shown that even a brief exposure (2 s) of the MagR-expressing hippocampal neurons to a magnetic field could induce a dramatic increase in [Ca^2+^]i that lasted for more than 10 s (Long et al., 2015). Curiously, unlike what was reported in HEK 293 cells, the calcium response in neurons exhibited a long delayed, up to 20 s (Long et al., 2015). In marked contrast to the above report, we observed no response to MS at up to 1.0 mT in MagR-expressing hippocampal neurons of various ages (Figure 2A). In either GCaMP6-P2A-MagR transfected neurons or neurons co-transfected with GCaMP6 and MagR (GCaMP6+MagR-mCherry), with either short (2 s) or long (25 s) duration of MS, no increase in intracellular calcium was observed up to 50 s after the termination of MS (Figures 2C,D). The negative control, cells expressing GCaMP6 alone also exhibited no calcium response after application of MS (Figure 2C). At the end of each experiment, we applied a high concentration of potassium ions (high K^+^, KCl, 50 mM) to induce depolarization in the cultured hippocampal neurons. Neuronal depolarization by high K^+^ induced a dramatic increase in GCaMP6 fluorescent signal (Figures 2B,D). In individual neurons, the calcium response to high K^+^ could vary from 100 to 500%, but no obvious differences could be detected in neurons transfected with GCaMP6, GCaMP6-P2A-MagR, or GCaMP6+MagR-mCherry. These results suggest that these transfected hippocampal neurons respond normally to external stimulation and exhibit calcium influx.

Mature hippocampal neurons often exhibited oscillations of their intracellular calcium after days in culture (see example trace in Figure 2E), due largely to spontaneous firing of action potentials. To determine whether MagR could regulate the spontaneous calcium oscillations, we applied magnetic field stimulation to the MagR-expressing neurons. Application of MS seemed to have no effect on the spontaneous calcium oscillations. The repeated calcium fluctuations were not phase-locked to either “on” or “off” of the magnetic field. The frequency and magnitude of the calcium oscillations were not modulated by the magnetic field in either direction (Figure 2E).

**Figure 3:**
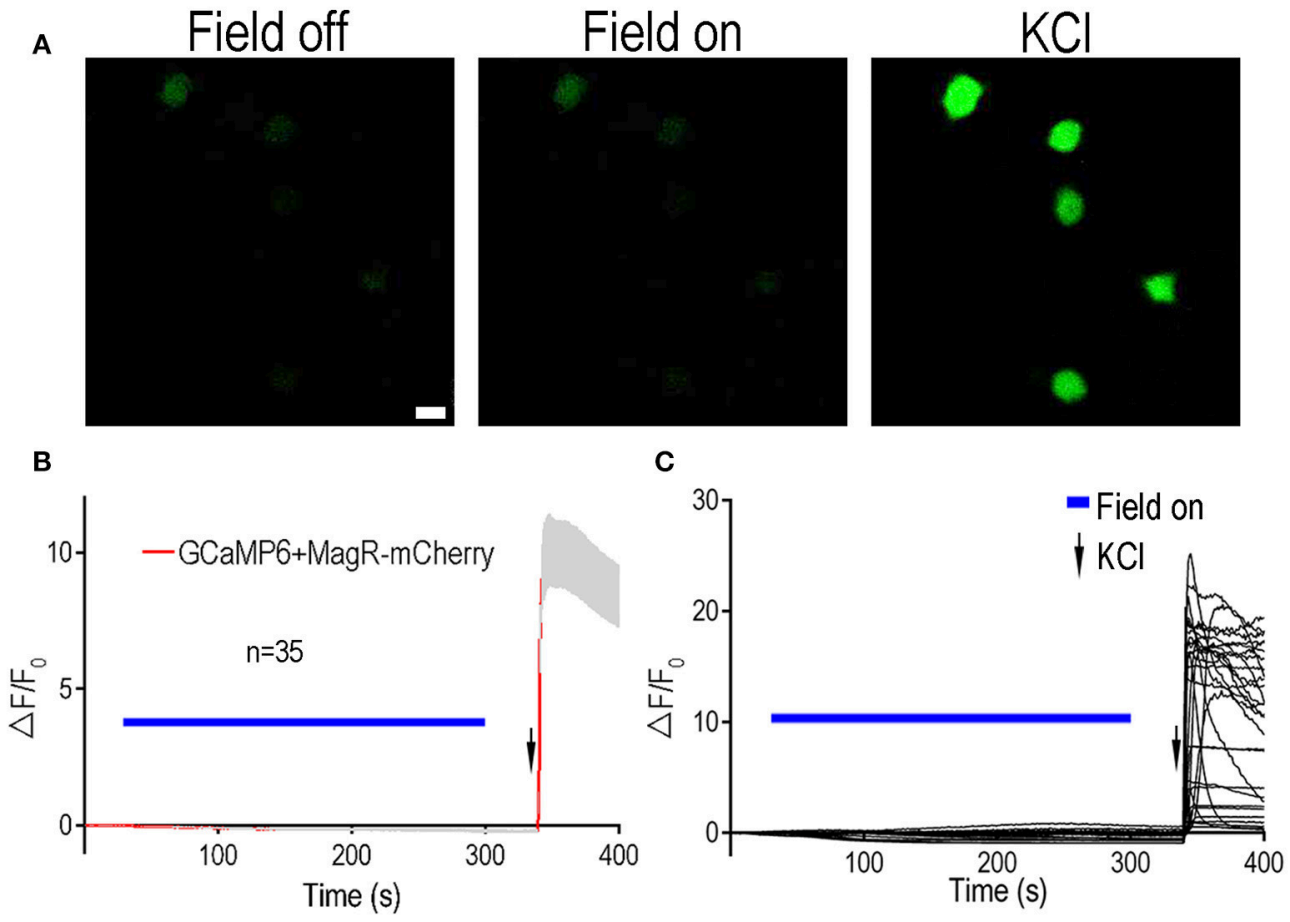
**Lack of Ca^**2+**^ responsiveness to magnetic stimulation in DRG neurons expressing MagR**. All procedures were the same as Figure 2 except dorsal root ganglion (DRG) neurons were electroporated with MagR-mCherry and GCaMP6. **(A)** Representative images showing the fluorescent intensity in the absence (Field off) and presence (Field on) of magnetic stimulation, and after applying high KCl. Scale bar: 20 μm. **(B)** 1.0 mT magnetic field failed to activate DRG neurons expressing MagR. Application of MS elicited no calcium response in the MagR expressing DRG neurons. However, high KCl induced dramatic elevation in ΔF/F_0_. Magnetic stimulation was indicated by blue bars above the curve, KCl application was indicated by arrows. *n* = 35. **(C)** Individual traces of recorded DRG neurons.

To further verify these results, we tested the effect of MagR on intracellular calcium changes in another type of neuron: rat dorsal root ganglion (DRG) neurons (Figure 3). Rat DRGs were dissected from both cervical and lumbar regions and dissociated. Both MagR-mCherry and GCaMP6 plasmids were co-transfected into the DRG neurons by electroporation (Huang and Neher, 1996). MS up to 1.0 mT did not change GCaMP6 fluorescence in transfected DRG neurons, which displayed obvious [Ca^2+^]i increase after the application of high concentration of K^+^ solution (Figure 3B). Increasing the duration of MS from 5 s to 2 min also failed to induce any significant changes (data not shown). In addition to static magnetic fields, we also used alternating magnetic fields, with the frequency varying from 2 to 5 Hz. Under no circumstance could we evoke any change in GCaMP6 fluorescence in DRG neurons (data not shown), suggesting stable [Ca^2+^]i. Taken together, expression of MagR alone in mammalian central or peripheral neurons did not confer any calcium responsiveness to MS, contrary to the previous report (Long et al., 2015).

### Fura-2 based ratiometric Ca^2+^ imaging

**Figure 4:**
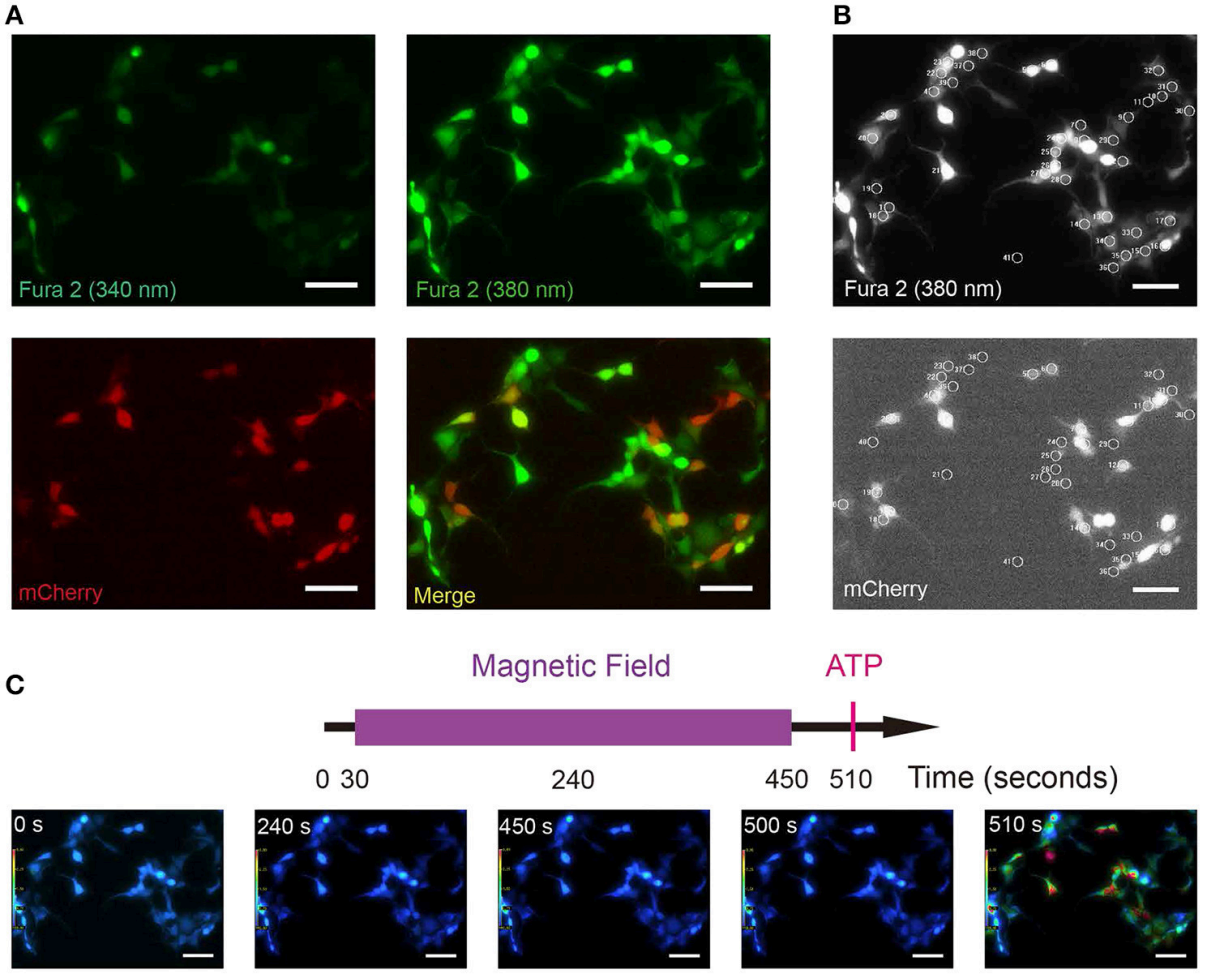
**Fura-2 based ratiometric Ca^**2+**^ imaging. (A)** Sample images of Fura-2 at 340, 380 nm wavelength illuminations (green, all cells) and mCherry to show the MagR-expressing cells (red). HEK 293A cells were transfected with MagR-IRES-mCherry, and stimulated by magnetic field. Intracellular Ca^2+^ changes were monitored by Fura-2 340/380 ratiometric imaging. **(B)** Upper: an example of Fura 2 (380 nm) image correspondent to 4A. Cells with 20 μm diameter circles were those used for 340/380 ratio imaging. Lower: Transfected and non-transfected cells (mCherry positive and negative) are highlighted by 20 μm diameter circles. **(C)** Top, timeline of calcium image protocol. Purple bar represents the period that the magnetic field stimulation was applied. The red vertical line represents application of ATP at the end of the experiment, as a control for cell viability. Bottom, 340/380 ratio false-color map at 0, 180, 450, 500, and 520 s. The warmer color represents the higher ratio of 340/380 and the higher calcium concentration. Scale bar, 100 μm.

Compared with GCamP6 intensity (ΔF/F0) which measures relative changes in [Ca^2+^]i, Fura-2 ratio (340/380) could measure absolute [Ca^2+^]i, avoiding the influence of photo-bleaching during recording. To exclude any potential artifacts due to a particular setup or system, and to replicate and validate the GCamP6-based findings in independent assays and systems, we repeated the above experiments using Fura-2 ratiometric single-cell calcium imaging in a different setup. We transfected HEK 293A cell line with the plasmid MagR-IRES-mCherry (Figures 4A,B). These cells were incubated in Fura-2-AM (Fura-2-acetoxymethyl ester), a membrane permeable, ratiometric calcium indicator whose acetoxymethyl groups are removed by cellular esterases, keeping it inside the cells. In our experiment, emission lights triggered by 340 and 380 nm LED illuminations were recorded separately, and the ratio of emission lights 340/380 was represented by pseudo-color (Figure 4). With this technique, we could simultaneously measure intracellular calcium concentrations in MagR (mCherry) -positive and –negative cells in the same field (Figure 4A). Transfected and non-transfected cells were selected with 20 μm diameter circles (Figure 4B). As shown in Figure 4C, [Ca^2+^]i before (0 s) and after (180, 450, and 500 s) MS were essentially the same. As a positive control, we applied ATP at the time point of 510 s. A dramatic increase in [Ca^2+^]i was observed in both MagR –positive and –negative cells (Figure 4C), indicating typical calcium response in these cells.

### Effect of magnetic field on intracellular Ca^2+^ concentration in 293 cells expressing MagR alone

**Figure 5:**
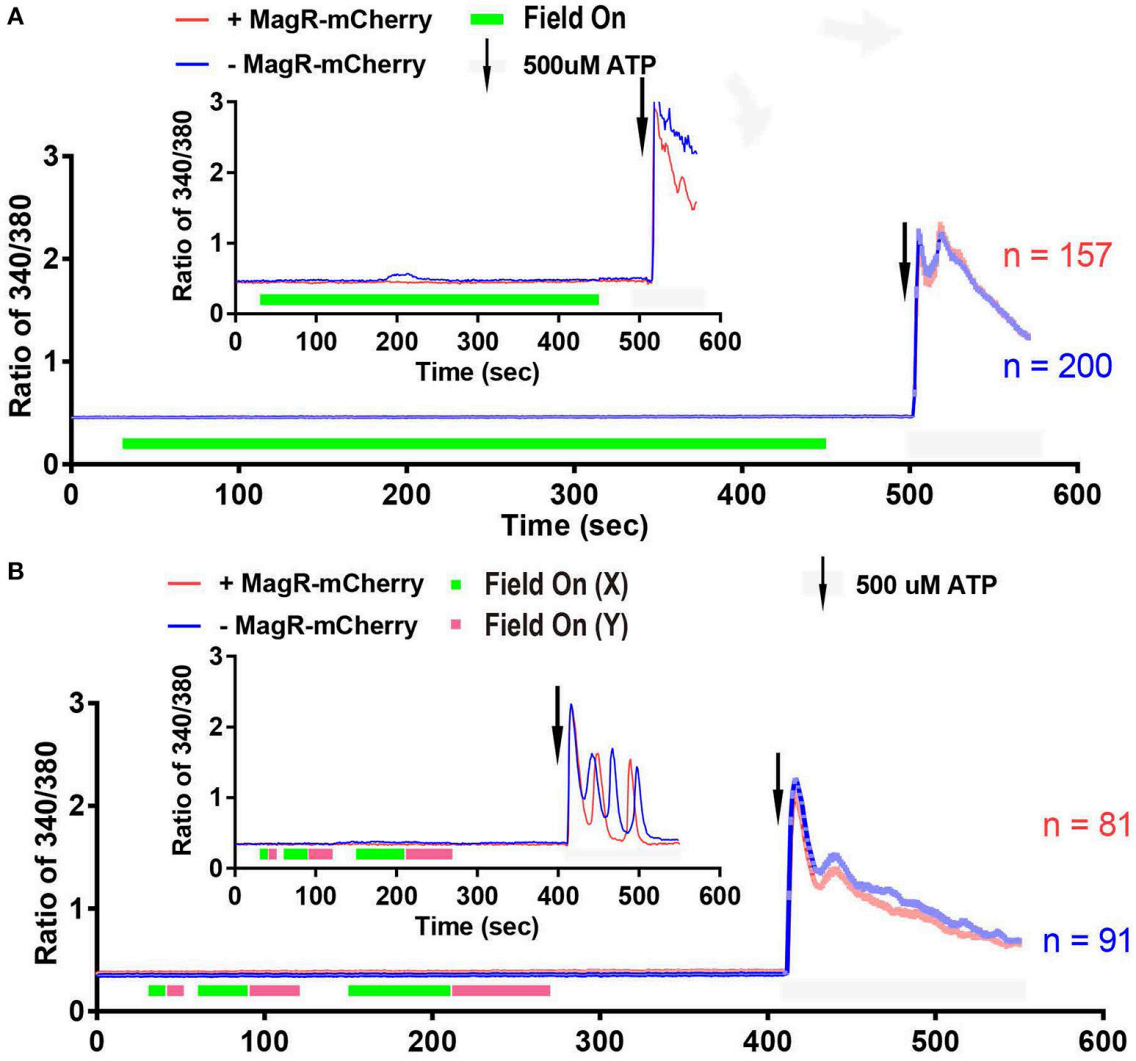
**Fura-2 based imaging failed to detect Ca^**2+**^ response to magnetic field in MagR-expressing 293 cells. (A)** Fura-2 ratiometric single-cell Ca^2+^ imaging of MagR-IRES-mCherry transfected 293A cells upon a 1.2 mT magnetic field stimulation in one direction. Note that there is no change in intracellular Ca^2+^ concentration over a 7-min period. **(B)** Response to 10, 30 and 60 s alternations of magnetic fields in two orthogonal directions (X and Y). Note that intracellular Ca^2+^ concentration does not change regardless of on/off, direction, or the duration of magnetic field stimulations. 500 μM ATP was applied at the end as a control for cell viability. Smaller insets in A and B are data from two pairs of randomly selected transfected and non-transfected cells.

We next performed a systematic examination of [Ca^2+^]i response in a large number of 293A cells. Quantitative analysis of data from hundreds of cells revealed no increase in 340/380 ratio upon application of the magnetic field in MagR-positive (*n* = 157) and MagR-negative (*n* = 200) cells (Figure 5A). The magnetic field was applied up to 7 min. In a small number of the MagR-positive and -negative cells, the 340/380 ratio exhibited a spontaneous fluctuation of [Ca^2+^]i in the range of 0.4–0.6 (Figure 5A inset: an example of the data recorded from a pair of single cells), but these changes had no correlation with either application of magnetic field or expression of MagR. The small rise and fall, or [Ca^2+^]i oscillation, might possibly be due to a subtle change in surrounding temperature or dynamic cellular microenvironment. As a positive control, the cells were perfused with ATP (final concentration, 500 μM) at the end of each trial (Figure 5A). The 340/380 ratio dramatically increased, indicating that the cells were capable of changing [Ca^2+^]i when given the right stimuli.

The previous paper Long et al. also reported that cells may respond when the direction of magnetic field was altered (Long et al., 2015). With the same magnetic device, we applied to MagR-transfected 293A cells the magnetic field with two orthogonal directions (X and Y) sequentially for 10, 30, and 60 s (Figure 5B). The 340/380 ratio was unchanged in either short-duration alternation (10 s) or in comparatively long-duration alternations (30 and 60 s) of the magnetic fields. Again, calcium response increased markedly upon application of ATP.

**Figure 6:**
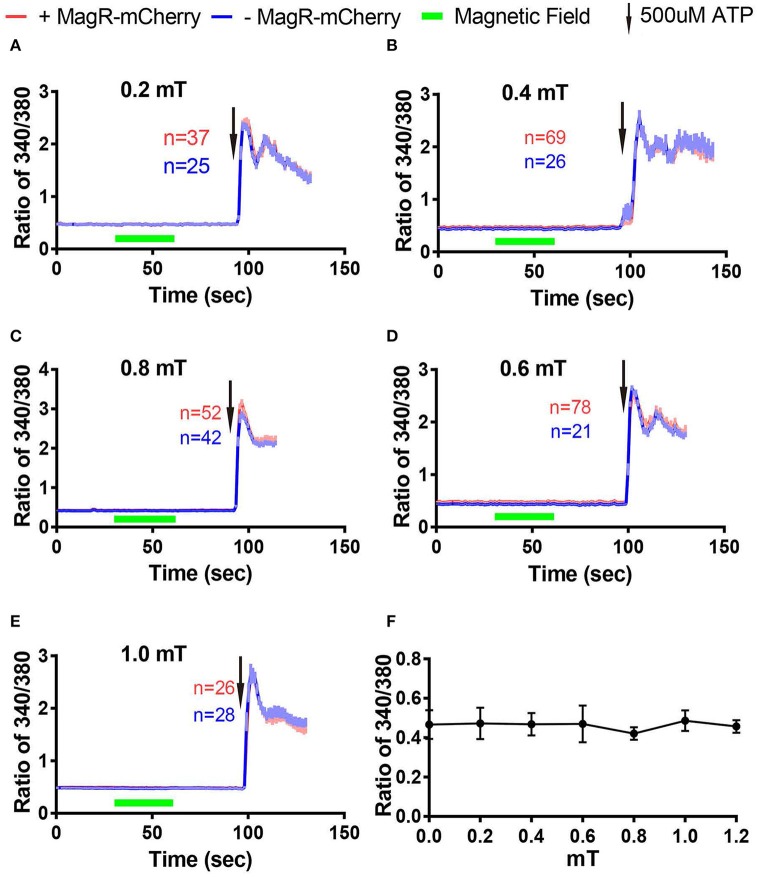
**Stimulation-response curves in 293 cells using Fura-2 imaging. (A–E)** Fura-2 ratiometric Ca^2+^ imaging of 293A cells transfected with MagR-IRES-mCherry responding to 0.2 mT **(A)**, 0.4 mT **(B)**, 0.6 mT **(C)**, 0.8 mT **(D)**, and 1.0 mT **(E)** magnetic field stimulations for 30 s. No change in Ca^2+^ concentration in any of the stimulation strengths tested. 500 μM ATP was applied at the end of experiments. **(F)** Summary of the effect of 27-s magnetic stimulation of various strengths on 293A cells transfected with MagR.

Next we examined whether magnetic field of different strengths could alter [Ca^2+^]i in Fura-2-AM ratiometric assay. The change of ratio 340/380 at 27 s after turning on of magnetic field was plotted against the strengths of magnetic fields measured at the center of the culture dish (Figure 6F). We found that [Ca^2+^]i failed to change in response to MS at 0.2, 0.4, 0.6, 0.8, and 1.0 mT (Figures 6A–E).

### Absence of change in intracellular Ca^2+^ response to magnetic field in hippocampal neurons expressing MagR alone

**Figure 7:**
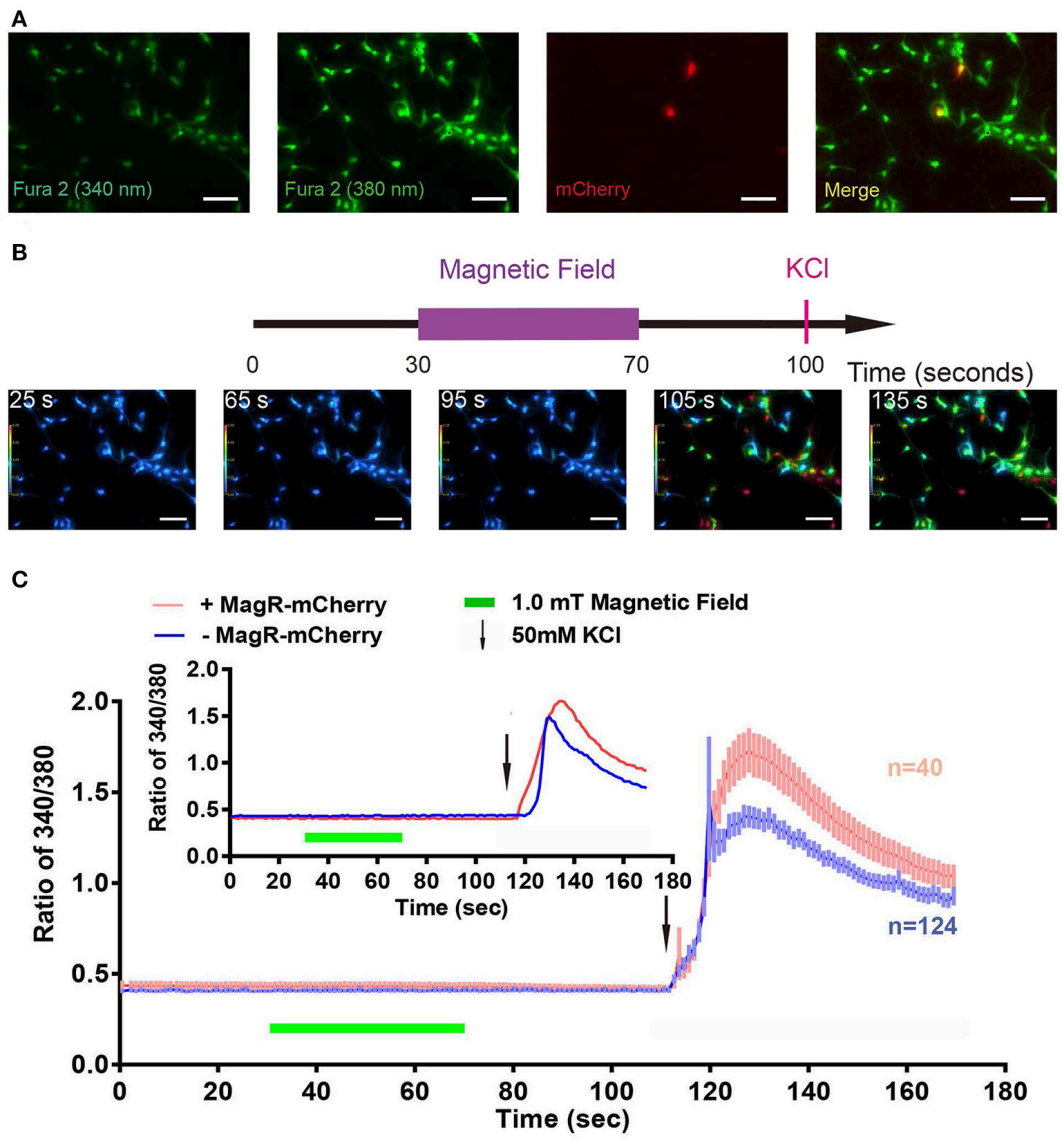
**Failure of magnetic stimulus to induce Ca^**2+**^ response in MagR-expressing hippocampal neurons**. Rat hippocampal neurons (DIV5~7) were transfected with MagR-IRES-mCherry, cultured for additional 24 h before processed for Fura-2 based Ca^2+^ imaging. **(A)** Fluorescence images of 340 nm, 380 nm, mCherry, and the merge of all three. **(B)** Top, timeline of Ca^2+^ imaging protocol. Purple bar represents the period that the magnetic field stimulation was applied. Red vertical line represents application of KCl at the end as a positive control. Bottom, 340/380 ratio false-color map at 25, 65, 95, 105, and 135 s. The warmer color represents the higher ratio of 340/380 ratio and the higher calcium concentration. **(C)** Fura-2 ratiometric Ca^2+^ imaging of MagR-IRES-mCherry transfected hippocampal neurons respond to 1.0 mT magnetic field for 40 s in one direction. No change in intracellular Ca^2+^ concentration was detected during this period. Smaller inset is the data of one pair of randomly selected transfected and non-transfected cells. Scale bar, 100 μm.

We also used the Fura-2-AM ratiometric assay to examine whether MS could change [Ca^2+^]i in MagR-expressing hippocampal neurons. Cultured neurons from hippocampus were transfected with MagR-IRES-mCherry on DIV6, and the ratiometric assay was conducted 24 h later. The transfection rate of MagR (indicated by mCherry-expressing neurons) was ~1% (Figure 7A). MS was applied to the whole culture dish, and MagR-positive and -negative neurons were simultaneously recorded. [Ca^2+^]i before (25 s) and after (65 and 90 s) MS were essentially the same (Figure 7B). Quantitative analysis showed no change in [Ca^2+^]i when the magnetic field was turned on or off, or during the entire course of MS in MagR-positive (*n* = 40) and MagR-negative (*n* = 124) neurons (40 s, Figure 7C). Occasionally, we observed some rise and fall of [Ca^2+^]i in a small number of neurons. However, these changes occurred in both MagR-positive and -negative neurons, and did not correlate with the on or off state of magnetic field stimulation (Supplemental Figure 2). We applied high K^+^ (50 mM) to induce neuronal depolarization at the end of each experiment (105 s time point). A dramatic increase in ratio of 340/380 was observed in both MagR –positive and –negative neurons, ranging from about 0.4–1.5. These results suggest that calcium influx in these neurons was normal, that the assay worked, and that the neurons were healthy.

To ensure that the cultured hippocampal neurons were fully competent in exhibiting calcium influx in responding to external stimuli, we used optogenetics to activate the neurons. Hippocampal neurons co-transfected the calcium indicator RCaMP as well as channelrhodopsin2 (ChR2)-YFP-P2A-MagR. As shown in Supplemental Figure 4, application of MS to the Mag-R and ChR2 co-transfected neurons resulted in no change in intracellular calcium (RCaMP signal). Subsequent application of blue light (471 nm laser stimulation) to the very same neurons induced a marked increase in ΔF/F_0_ (Supplemental Figure 4). Application of high K^+^ (50 mM) at the end of the experiment also elicited a large calcium response (Supplemental Figure 4). Similar experiments were performed several times and the same results were obtained (*n* = 3). Taken together, these results strongly argue against the possibility that MS through MagR alone could induce intracellular calcium signaling.

## Discussion

With the advances in optogenetics, a growing interest in the field of neuromodulation is to develop new technologies that could overcome the limitations of light stimulation in modulating neuronal activities. Magnetic field stimulation (MS) has obvious advantages in its non-invasiveness, deep penetration and long-distance action. A series of prominent papers have been published recently, showing the use of magnetic field-sensing proteins to activate neurons (Stanley et al., 2016; Wheeler et al., 2016). In particular, Long et al. claimed that expression of MagR as a standalone tool renders HEK293 cells and hippocampal neurons responsive to MS with a power density as low as 1.0 mT (Long et al., 2015). To systematically evaluate the utility of MagR, we focused on calcium responses in MagR-expressing cells. Initially we used conditions almost identical to those used in the Long report, including MagR alone plus GCaMP6 and MagR-P2A-GCaMP6 constructs that Long et al had used (data not shown). We then extended our investigations to different types of cells (HEK293A and HEK293T cells, hippocampal neurons, or dorsal root ganglion neurons), different stimulation protocols (long/short, on/off, X/Y axis, different power densities), different ways of measuring calcium responses (GCaMP6, Fura-2 AM), and different DNA constructs and transfection methods. Immunostaining and Western blots were performed using a newly generated monoclonal antibody against MagR to ensure the expression of MagR in transfected cells. At the end of each recording, we applied agents known to elevate intracellular calcium as positive controls to ensure that cells are healthy and can exhibit calcium responses. With numerous repetitions in multiple labs and setups, we could not escape the conclusion that MS in our hands cannot induce any calcium responses in any types of cells expressing MagR alone. In some cases, we co-expressed MagR and channelrhodopsin and showed that the same neurons incapable of responding to MS can indeed be activated by light. These findings cast serious doubts on the previous claim that MagR alone could mediate neuronal activation in response to MS. Our results should also help other labs to conduct future studies in the field.

### Calcium response in MagR-expressing HEK293 cells

An intriguing result reported by Long et al. was that MS induced a huge calcium influx, a 350% increase over baseline, in HEK293A cells expressing MagR (Long et al., 2015). While it was not described clearly how the MS was applied, it seems that the magnetic stimulus was turned on for as long as 7 min. We conducted similar experiments, applying magnetic stimulus to the same cell line by the same homemade device for the same duration. In marked contrast, we did not observe any change in cellular calcium with or without MS. The only difference was that we used an inverted microscope with ample air circulation while the previous study used an upright microscope. One cannot rule out the possibility that the lens of the upright microscope impeded thermal dispersion so that the heat generated by the magnetic device could raise the temperature of the cultured cells, leading to calcium changes.

Consistent with this interpretation, Long et al. showed that the calcium signal in the MagR-expressing HEK293 cells continued to rise but never came down even after the termination of MS (Figures 1E, 2C, Long et al., 2015). A sustained elevation of intracellular calcium could be an indication of an unhealthy state of the responding cells. It is therefore possible that the increase in intracellular calcium observed by Long et al. (2015) was due to unhealthy cell state (poor culture, improper DNA transfection, temperature fluctuation, etc.), and not by MS. In each of our experiments, we applied ATP, an agent known to induce calcium elevation in HEK293 cells. We invariably observed a robust increase in intracellular calcium upon ATP application, followed by a rapid decline, indicating that these cells remained healthy. Our results do not support the hypothesis that MagR alone is sufficient to mediate calcium influx in response to magnetic field stimulation in HEK293 cells.

### Calcium response in MagR-expressing hippocampal neurons

In multiple labs using different setups, we failed to observe any change in intracellular calcium upon MS in MagR-expressing hippocampal neurons or DRG neurons. A variety of stimulation protocols were used including turning MS on and off, applying MS for a short or long duration, and switching MS from X-axis to Y-axis. Under no circumstance did we see any effect of MagR expression alone. This is again in marked contrast to the paper by Long et al. (2015) who reported a robust calcium influx in MagR-expressing hippocampal neurons upon MS. It is well known that hippocampal neurons in culture exhibit spontaneous firings, leading to oscillations of intracellular calcium. Indeed, we observed occasionally fluctuation of [Ca^2+^]i in our recordings, possibly due to extensive synaptic connections (Supplemental Figure 1). However, the rise and fall also occurred in MagR-negative neurons, and did not follow MS (Supplemental Figure 1), suggesting that these [Ca^2+^]i oscillations were caused neither by MagR nor by MS.

Peculiarly, the MS-induced calcium influx in hippocampal neurons observed by Long et al. occurred with a very long delay, as long as 7.8 s after the onset of MS (Long et al., 2015). This is very unusual, because all neuronal stimuli reported so far, electrical, chemical, light, mechanical, etc., fall in the millisecond range. Given that neurons exhibit spontaneous firings at random, it is difficult not to question whether the change in [Ca^2+^]i reported by Long et al. (2015) was merely random firing of the cultured neurons. A series controls using sodium channel blocker tetrodotoxin, glutamate transmission blockers CNQX/Apv would have helped to rule out the potential artifacts due to spontaneous neuronal firing and glutamate transmission. Regardless, these analyses have raised serious questions about whether MagR alone could be used for magnetogenetics.

In conclusion, the discovery that MagR/Cry is a putative magneto-responsive protein complex do not directly imply that MagR itself may induce neuronal response in transfected cells. While the possibility exists that MagR, when associated with other proteins such as Cry or linked to other channels such as TRV4 may be used for magnetogenetics, our present results suggest that more factors seem necessary, in addition to expression of MagR alone, for MagR to be used as a tool for neuronal modulation via magnetic field. We thus urge more studies in this regard to fully uncover the underlying molecular mechanisms of MagR/Cry mediated magnetoreception and the coupling between light- and magneto-receptions, so that promising magnetogenetic applications may be developed.

## Author contributions

PC, KP, and BL initiated the project after discussion with CX. BL, WG, and KP designed the study. KP, HY, YC, PC, MH, and JS conducted the experiments and analyzed the data. BL, HY, YC, and KP wrote the manuscript.

### Conflict of interest statement

The authors declare that the research was conducted in the absence of any commercial or financial relationships that could be construed as a potential conflict of interest.

## Supplementary material

Supplemental Figure 1 **Sample images of cells co- transfected of with MagR-IRES-mCherry and GcAMP6S**. HEK293 cells were immunostained with a mouse monoclonal anti-MagR antibody, followed by Alexa Fluor647 donkey anti-mouse IgG secondary antibody (excitation wavelength 647 nm). Upper: To validate that Anti-MagR antibody specifically stained MagR-expressing cell, HEK293 cells were transfected with GCaMP6s only as negative control. Note that there is only GCaMP6s fluorescence but no MagR staining (purple) in any cells. Lower: HEK293 cells were co-transfected with GCaMP6s and MagR-IRES-mCherry. The anti-MagR antibody specifically stained co-transfected cells and did not stain cells without MagR expression. A merge view shows co-localization of MagR, mCherry and GCaMP6s. Scale bar = 10 μm.

Supplemental Figure 2 **An example of spontaneous firing of hippocampal neuron in the present of magnetic field**. HEK293T cells were transfected with only GCaMP6 but no MagR, and subjected to calcium imaging. Blue and magenta bars indicate application of magnetic field in one direction (X-axis) or another, perpendicular direction (Y-axis). Sporadic increases in Ca^2+^ fluorescence were seen, but they have no correlation with “on” or “off,” or the direction, of the MS.

Supplemental Figure 3 **Intracellular calcium fluctuated in the absence of magnetic field**. Cells were transfected with GCaMP6 only, and calcium signals were recorded over time in the absence of magnetic field stimulation. In two representative cells indicated by the green and red arrows in inset, one (red) exhibited no change in calcium fluorescence while the other showed a small fluctuation at around the 20 s time point.

Supplemental Figure 4 **Magnetic and optical stimulation of the same neuron**. Hippocampal neurons were co-transfected with Mag-R and ChR2. Magnetic and optical stimulation are indicated by blue and cyan bars above the curve, respectively. Light but not magnetic stimulation applied to the same cells induced an increase in calcium signals. The black arrow marks the application of KCl, which induced a large calcium response.
