# Supplementary figures and images for: MagR Alone Is Insufficient to Confer Cellular Calcium Responses to Magnetic Stimulation
Source: Front Neural Circuits. 2017 Mar 16;11:11. doi: 10.3389/fncir.2017.00011 (PMC5352684; doi:10.3389/fncir.2017.00011)

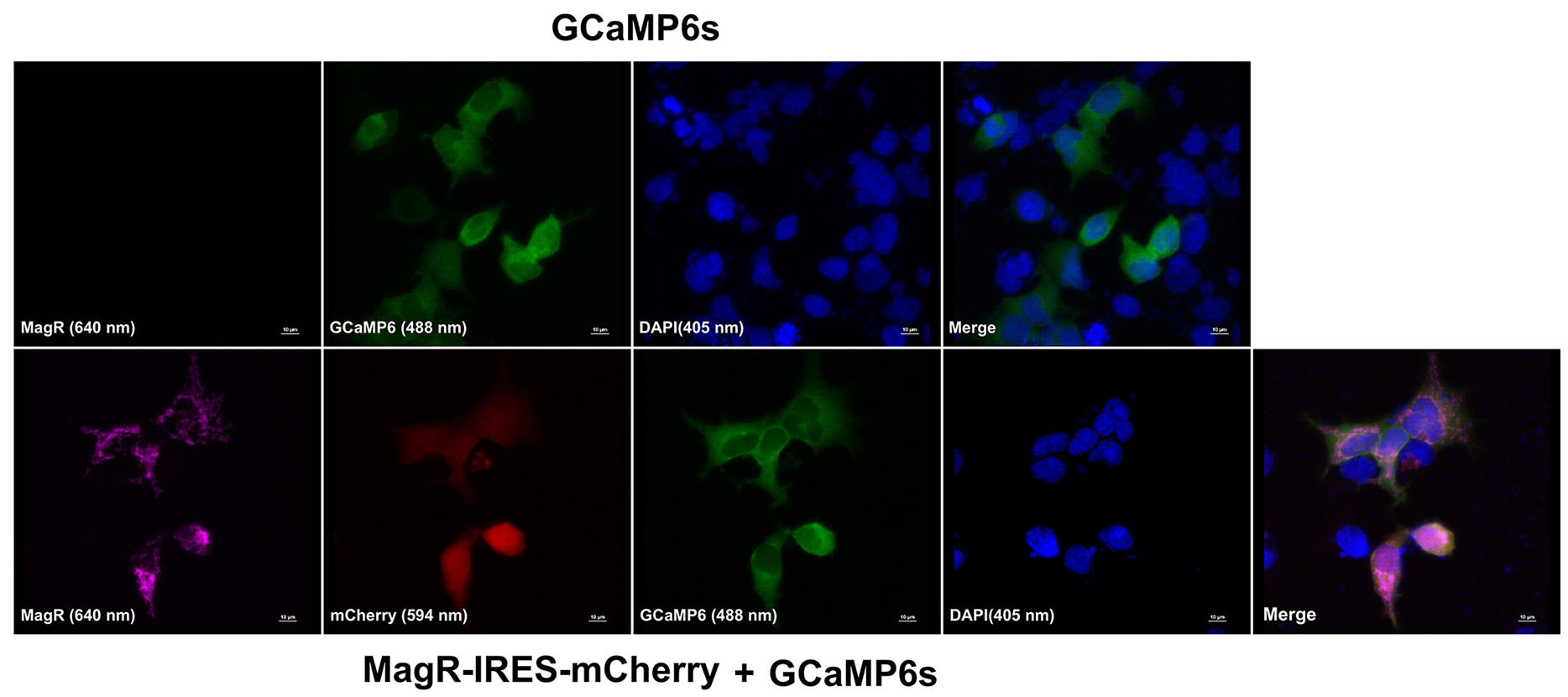

Supplement: Supplemental Figure 1 — Sample images of cells co- transfected of with MagR-IRES-mCherry and GcAMP6S. HEK293 cells were immunostained with a mouse monoclonal anti-MagR antibody, followed by Alexa Fluor647 donkey anti-mouse IgG secondary antibody (excitation wavelength 647 nm). Upper: To validate that Anti-MagR antibody specifically stained MagR-expressing cell, HEK293 cells were transfected with GCaMP6s only as negative control. Note that there is only GCaMP6s fluorescence but no MagR staining (purple) in any cells. Lower: HEK293 cells were co-transfected with GCaMP6s and MagR-IRES-mCherry. The anti-MagR antibody specifically stained co-transfected cells and did not stain cells without MagR expression. A merge view shows co-localization of MagR, mCherry and GCaMP6s. Scale bar = 10 μm. [file Image1.JPEG]

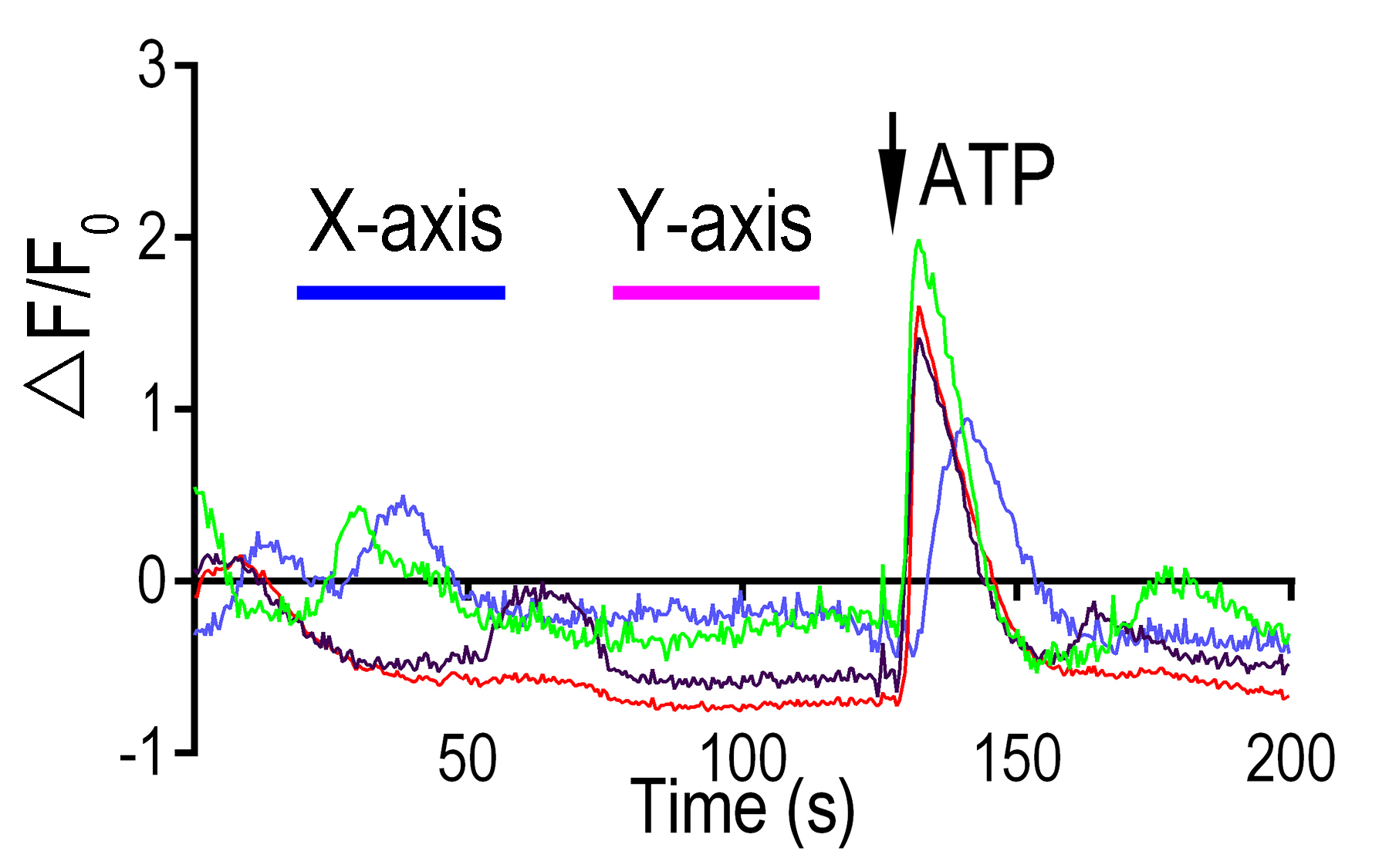

Supplement: Supplemental Figure 2 — An example of spontaneous firing of hippocampal neuron in the present of magnetic field. HEK293T cells were transfected with only GCaMP6 but no MagR, and subjected to calcium imaging. Blue and magenta bars indicate application of magnetic field in one direction (X-axis) or another, perpendicular direction (Y-axis). Sporadic increases in Ca2+ fluorescence were seen, but they have no correlation with “on” or “off,” or the direction, of the MS. [file Image2.JPEG]

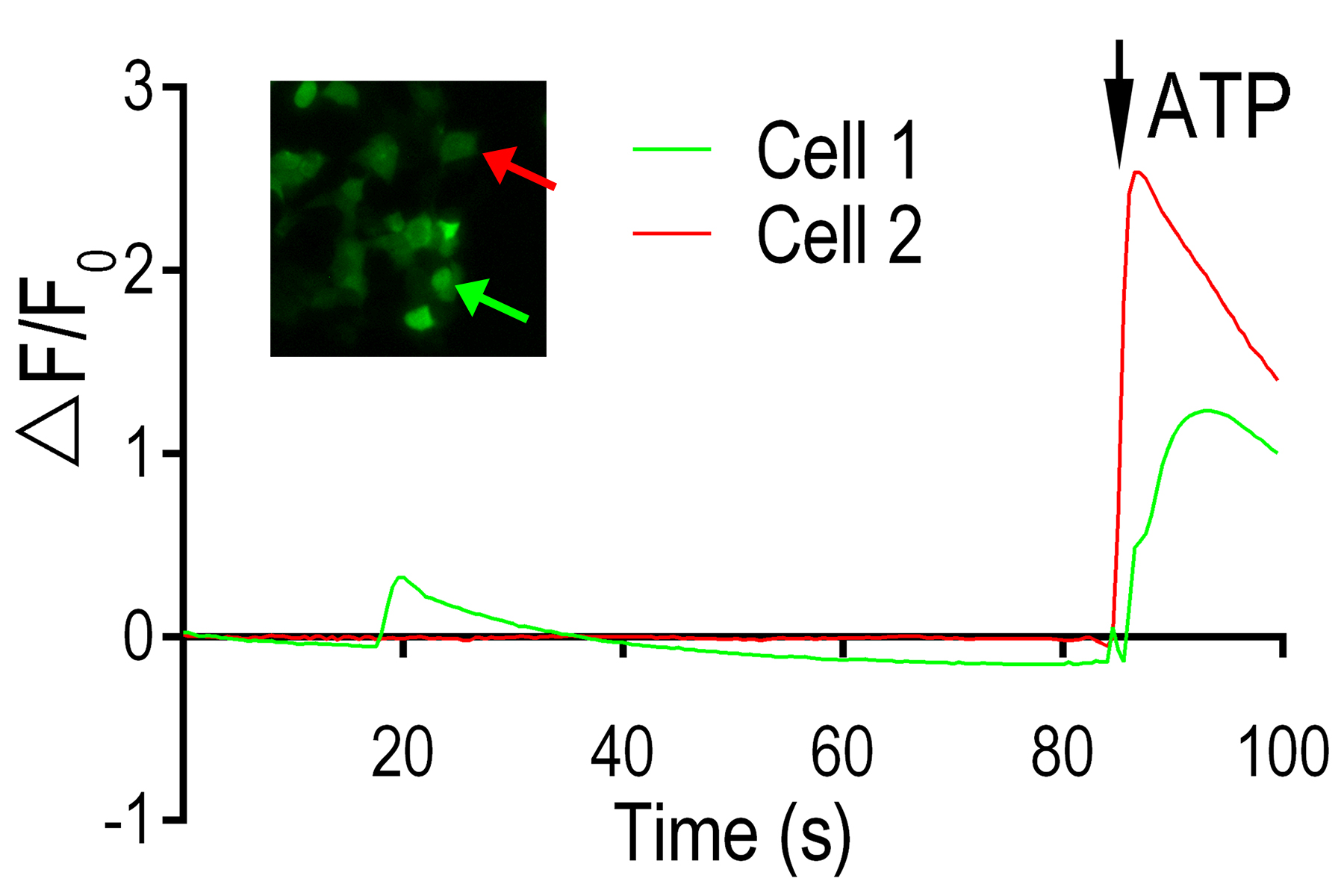

Supplement: Supplemental Figure 3 — Intracellular calcium fluctuated in the absence of magnetic field. Cells were transfected with GCaMP6 only, and calcium signals were recorded over time in the absence of magnetic field stimulation. In two representative cells indicated by the green and red arrows in inset, one (red) exhibited no change in calcium fluorescence while the other showed a small fluctuation at around the 20 s time point. [file Image3.JPEG]

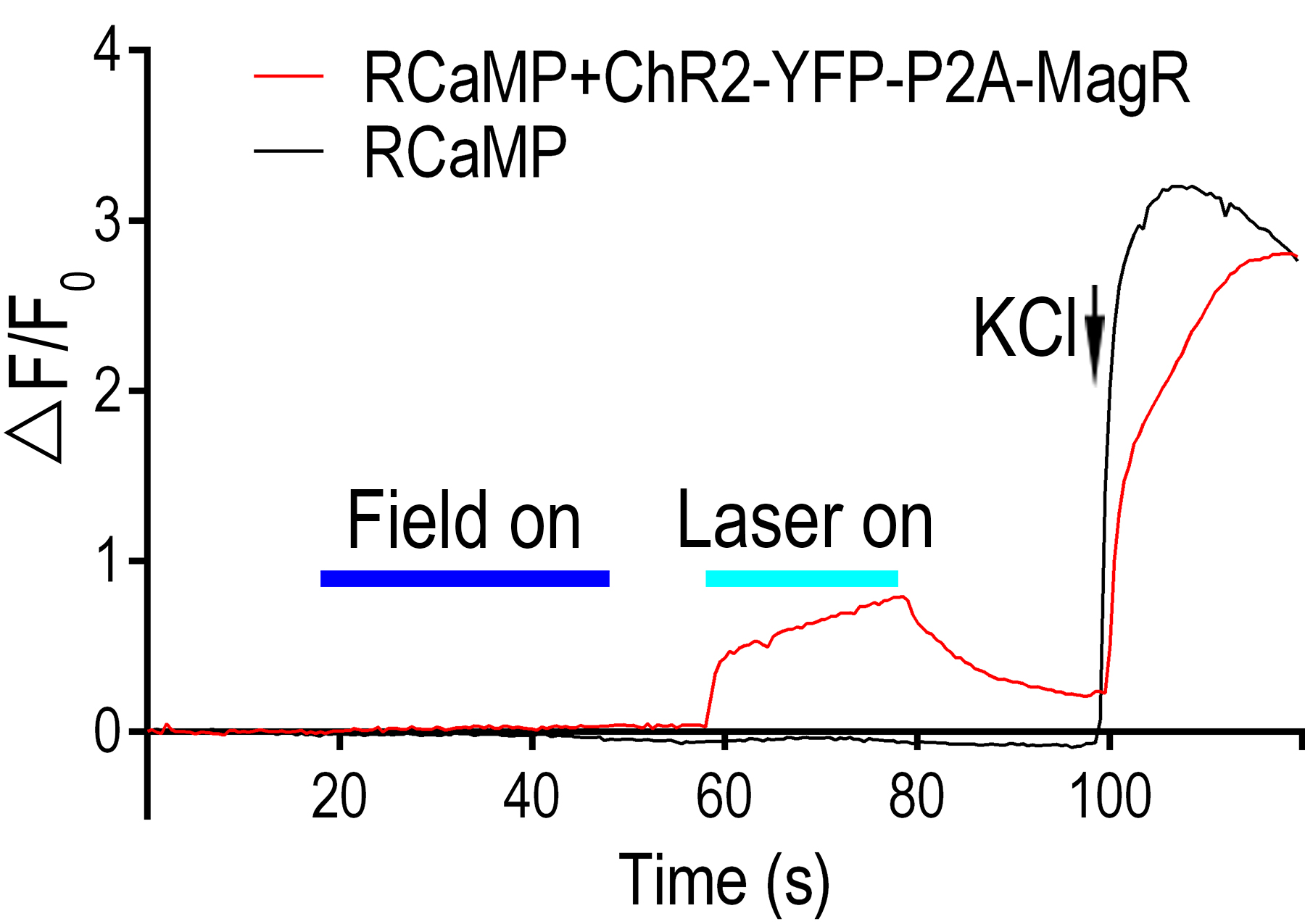

Supplement: Supplemental Figure 4 — Magnetic and optical stimulation of the same neuron. Hippocampal neurons were co-transfected with Mag-R and ChR2. Magnetic and optical stimulation are indicated by blue and cyan bars above the curve, respectively. Light but not magnetic stimulation applied to the same cells induced an increase in calcium signals. The black arrow marks the application of KCl, which induced a large calcium response. [file Image4.JPEG]
